# Barriers and facilitators to person-centred infection prevention and control: results of a survey about the Dementia Isolation Toolkit

**DOI:** 10.1186/s12877-022-02759-4

**Published:** 2022-01-25

**Authors:** Andrea Iaboni, Hannah Quirt, Katia Engell, Julia Kirkham, Steven Stewart, Alisa Grigorovich, Pia Kontos, Josephine McMurray, AnneMarie Levy, Kathleen Bingham, Kevin Rodrigues, Arlene Astell, Alastair J. Flint, Colleen Maxwell

**Affiliations:** 1 KITE-Toronto Rehabilitation Institute, University Health Network, Toronto, Ontario Canada; 2 Department of Psychiatry, Temerty Faculty of Medicine, University of Toronto, Toronto, Ontario Canada; 3 Department of Psychiatry, Cumming School of Medicine, University of Calgary, Calgary, Ontario Canada; 4 Department of Community Health and Epidemiology, Dalhousie University, Halifax, Nova Scotia Canada; 5 Department of Recreation and Leisure Studies, Faculty of Applied Health Sciences, Brock University, St. Catherines, Ontario, Canada; 6 Dalla Lana School of Public Health, University of Toronto, Toronto, Ontario Canada; 7 Lazaridis School of Business & Economics, Wilfrid Laurier University, Brantford, Ontario Canada; 8 Centre for Mental Health, University Health Network, Toronto, Ontario Canada; 9 Departments of Occupational Sciences & Occupational Therapy and Psychiatry, University of Toronto, Toronto, Ontario Canada; 10 School of Psychology & Clinical Language Sciences, University of Reading, Reading, UK; 11 Schools of Pharmacy and Public Health & Health Systems, University of Waterloo, Waterloo, Ontario Canada

## Abstract

**Background:**

People working in long-term care homes (LTCH) face difficult decisions balancing the risk of infection spread with the hardship imposed on residents by infection control and prevention (ICP) measures. The Dementia Isolation Toolkit (DIT) was developed to address the gap in ethical guidance on how to safely and effectively isolate people living with dementia while supporting their personhood. In this observational study, we report the results of a survey of LTCH staff on barriers and facilitators regarding isolating residents, and the impact of the DIT on staff moral distress.

**Methods:**

We completed an online cross-sectional survey. Participants (n = 207) were staff working on-site in LTCH in Ontario, Canada since March 1, 2020, with direct or indirect experience with the isolation of residents. LTCH staff were recruited through provincial LTCH organizations, social media, and the DIT website. Survey results were summarized, and three groups compared, those: (1) unfamiliar with, (2) familiar with, and (3) users of the DIT.

**Results:**

61% of respondents identified distress of LTCH staff about the harmful effects of isolation on residents as a major barrier to effective isolation. Facilitators for isolation included delivery of 1:1 activity in the resident’s room (81%) and designating essential caregivers to provide support (67%). Almost all respondents (84%) reported an increase in moral distress. DIT users were less likely to report an impact of moral distress on job satisfaction (odds ratio (OR) 0.41, 95% CI 0.19-0.87) with 48% of users reporting the DIT was helpful in reducing their level of moral distress.

**Conclusions:**

Isolation as an ICP measure in LTCH environments creates moral distress among staff which is a barrier to its effectiveness. ICP guidance to LTCH would be strengthened by the inclusion of a dementia-specific ethical framework that addresses how to minimize the harms of isolation on both residents and staff.

**Supplementary Information:**

The online version contains supplementary material available at 10.1186/s12877-022-02759-4.

## Background

Long-term care homes (LTCH) and other aged residential care settings have been devastated by the novel coronavirus COVID-19 pandemic. The pandemic has resulted in the death of approximately one in five infected residents [1], and the prolonged use of strict infection control and prevention (ICP) measures including separation from families and isolation to bedrooms. While isolation and quarantine are two highly effective strategies for slowing the spread of infectious diseases, achieving effective isolation is especially challenging when caring for residents with dementia who comprise approximately 70% of the LTCH population [2]. These residents may find it difficult to understand, remember, and follow restrictions imposed on their movements in the context of isolation [3]. Further, the resulting disruption to their care routines and social interactions while in isolation negatively impacts their mental and physical health and quality of life [4–7].

Strategies used to restrict the movements of residents can sometimes involve the use of restraint (either pharmacological management, physical restraint, or seclusion), placing LTCH residents at risk of serious harm [8–10]. These types of restrictive interventions are in conflict with the person-centred care principles that are used to guide the care of residents in LTCH. In the context of ICP, there are significant ethical tensions between supporting the safety of the wider community in the LTCH and minimizing the negative impacts of these policies on individuals [11, 12]. Ethical challenges such as these are known to produce moral distress in healthcare workers [13, 14]. However, the potential for moral distress was not acknowledged in the early public health and ICP directives to LTCH [15, 16]. These provincially-issued directives were required to be followed by all LTCH in Ontario and offered little leeway for local decision-making. No guidance was provided about how ICP directives could be applied while still practicing in accordance with person-centred care principles [17, 18].

To address these gaps, the Dementia Isolation Toolkit (DIT; www.dementiaisolationtoolkit.com) [19] was designed and developed in partnership with a number of LTCH stakeholders to address two aims: (1) To support the compassionate, safe and effective isolation and quarantine of residents of LTC; and (2) To support the moral resilience of LTCH staff. The toolkit includes plain-language ethical guidance specific to the use of isolation as an ICP measure in LTCH. This guidance sets out the principles of public health ethics and outlines the ethical duty of those imposing public health measures to mitigate their harms. There is an ethical decision-making tool to help apply these principles to specific ethically challenging situations. Finally, the DIT provides a person-centred isolation care-planning tool to guide the development of care plans to support individual residents during a period of isolation. Since its release online on April 23, 2020, the DIT has been downloaded more than 8000 times, and has been widely disseminated [1, 20, 21].

As part of the development and evaluation of the DIT, we conducted a survey of LTCH staff in Ontario about their experiences isolating residents and their use of the DIT. The objectives of this study were to identify the most important barriers and facilitators to isolating residents during an outbreak, and to conduct a preliminary evaluation of the use of the DIT and its impact on LTCH staff moral distress.

## Methods

### Survey

The results presented here are part of a larger survey about the impact of the COVID-19 pandemic on resident care and staff moral distress in LTCH. The survey was developed by the DIT research team in consultation with LTCH stakeholders. Moral distress items were adapted from a validated moral distress in dementia care survey to refer to specific pandemic-related challenges [22]. Feedback on the survey was provided by DIT LTCH sector partners (Behavioural Supports Ontario (BSO), Regional Geriatric Program, Ontario Long-Term Care Association, AdvantAge Ontario) and the Survey Research Centre (University of Waterloo). The survey questions were piloted with 5 LTCH front-line staff identified through the research team and our partners to test the clarity, ease of completion, and design of the survey. The survey questions can be found in Supplementary materials.

The survey was programmed and hosted online by the Survey Research Centre at the University of Waterloo. Web survey data collection took place from December 16, 2020 to March 12, 2021. The study was approved by the University Health Network Research Ethics Board (REB#20-5866) and a University of Waterloo Research Ethics Committee (ORE#42,803), and was conducted in accordance with the principles of the Declaration of Helsinki. Survey participants reviewed an online consent form, then provided informed consent by indicating they had reviewed this information and agreed to participate by clicking a button which directed them to the survey.

### Participants and recruitment

To be eligible to complete the survey, the respondent must have physically worked at a LTCH in Ontario, Canada during the first wave of the COVID-19 pandemic (i.e., after March 1, 2020) and be fluent in English. An open link to the survey was distributed to the DIT partners and by the research team via online newsletters, electronic mailing-lists, websites, networks, and social media. Approximately 4-6 weeks after the first invitation was distributed, reminders were sent out via these same methods. In addition, LTCH staff who downloaded the DIT from the study website and provided their email address were invited to complete the survey. Reminder emails were also sent twice to this group. A draw for one of five $100 gift cards was used as an incentive to complete the survey.

### Sample size

We determined that a final sample size of 185 would achieve 80% power and a sample of 213 would achieve 90% power to detect medium effect sizes (Cohen’s w of 0.30; alpha=0.05). The target sample size was thus set at 300 to accommodate exclusions and incomplete surveys.

### Analysis

The survey results were summarized descriptively, including proportions, and their confidence intervals calculated using a logit transform to ensure confidence interval endpoints were between 0 and 1 and then expressed as percentages.

After excluding any respondent who had not participated in the isolation or quarantine of residents, the sample for analysis was trisected into: (1) a group reporting no familiarity with the DIT; (2) a group who were familiar with the DIT, but had not used it; and (3) a group who had used the DIT. Differences between the three groups in their characteristics were compared using Pearson chi-squared tests and multinomial logistic regression. Ordered logistic regression was used to examine the differences in moral distress between groups, using unadjusted models and models adjusted for identified differences in characteristics between groups.

Respondents also provided free-text responses to open-ended questions, and these responses were analyzed using conceptual content analysis to establish the frequency of certain concepts within the responses [23, 24]. The text was reviewed by authors AI and HQ, broken down into units (sentences or phrases) and coded by concepts. The concepts were then grouped into three categories and illustrative quotes identified.

## Results

### Respondents

There were 228 completed surveys, 98 incomplete surveys (excluded from analysis), and 2421 dropouts. Of the dropouts, 2364 (98%) occurred on the consent page and likely represented individuals who had clicked the link in error, clicked on the survey link more than once or who did not meet the inclusion criteria to continue the survey. One survey was completed by a family member of a LTCH resident and was excluded from the analysis. The average survey completion time was 23 min.

Two hundred and seven (91%) of survey respondents reported participating directly or indirectly in the isolation and quarantine of residents and were included in this analysis (Table 1). The respondents are almost all female (90%) and white (76%). They were mostly experienced LTCH staff (75% with more than 5 years’ experience) and 18% were in administrative roles. 78% of respondents had experienced a COVID-19 outbreak at their LTCH.

**Table 1:** Descriptive characteristics of survey respondents who participated in the isolation or quarantine of LTCH residents (n (column %))

	Total (N = 207)	Not familiar with DIT (n = 72)	Familiar with the DIT (n = 70)	Used the DIT (n = 65)	Pearson chi2 (p-value)
**Participant characteristics**					
**Age**					9.3, *df*=6 (0.158)
18-34	55 (27%)	21 (29%)	21 (29%)	20 (31%)	
35-44	52 (25%)	23 (32%)	23 (32%)	13 (20%)	
45-54	56 (27%)	18 (25%)	18 (25%)	14 (22%)	
55 or older	44 (21%)	10 (14%)	10 (14%)	18 (28%)	
**Gender**					**6.8**, ***df*=2** **(0.033)**
Female	186 (90%)	61 (85%)	65 (93%)	60 (92%)	
Male	14 (7%)	9 (12%)	1 (1%)	4 (6%)	
Other / No answer	7 (3%)	2 (3%)	4 (6%)	1 (1.5%)	^c^
**Ethnicity**					2.2, *df*=2 (0.329)
White	158 (76%)	51 (71%)	57 (81%)	50 (77%)	
Other	49 (24%)	21 (29%)	13 (19%)	15 (23%)	
**Years of LTC experience**					**18.5**, ***df*=12** **(0.018)**
Less than 5 years	51 (25%)	25 (35%)	17 (19%)	9 (14%)	
6-10 years	52 (25%)	18 (25%)	10 (25%)	24 (37%)	
11-15 years	27 (13%)	9 (12%)	11 (13%)	7 (11%)	
16-20 years	24 (12%)	4 (6%)	10 (15%)	10 (15%)	
More than 20 years	53 (26%)	16 (22%)	22 (27%)	15 (23%)	
**Role in LTC**					**31.5**, ***df*=10** **(<0.001)**
Administrative/Management	38 (18%)	15 (21%)	15 (21%)	8 (12%)	
Front Line Nursing Staff^a^	31 (15%)	14 (19%)	11 (16%)	6 (9%)	
BSO leads^b^	62 (30%)	7 (10%)	21 (30%)	34 (52%)	
Allied Health	56 (27%)	28 (39%)	16 (23%)	12 (18%)	
Medical	9 (4%)	4 (6%)	3 (4%)	0	
Other	11 (5%)	4 (6%)	4 (5%)	3 (5%)	
**LTC home characteristics**					
**Number of beds**					3.0, *df*=8 (0.935)
Less than 50 beds	14 (7%)	4 (6%)	5 (7%)	5 (8%)	
50-99 beds	39 (19%)	14 (19%)	12 (17%)	13 (20%)	
100-149 beds	59 (29%)	23 (32%)	17 (24%)	19 (30%)	
150-199 beds	44 (21%)	14 (19%)	19 (27%)	11 (17%)	
More than 200 beds	50 (24%)	17 (23%)	17 (24%)	16 (25%)	
**Region**					9.1, *df*=4 (0.060)
Rural to small (<30,000)	46 (22%)	16 (22%)	14 (20%)	16 (25%)	
Medium (30,000 - <100,000)	61 (30%)	25 (35%)	26 (37%)	10 (16%)	
Large urban (>100,000)	99 (48%)	31 (43%)	30 (43%)	38 (59%)	
**Ownership status**					5.2, *df*=6 (0.514)
Municipal	59 (29%)	18 (25%)	24 (34%)	17 (27%)	
Not-for-profit	65 (32%)	27 (38%)	18 (26%)	20 (31%)	
For-profit	73 (35%)	22 (31%)	26 (38%)	25 (39%)	
I do not know	9 (4%)	5 (7%)	2 (3%)	2 (3%)	^c^
**COVID-19 outbreaks**					3.3, *df*=4 (0.511)
Zero	45 (22%)	13 (18%)	15 (21%)	17 (27%)	
One or two	88 (42%)	31 (43%)	34 (49%)	23 (36%)	
Three or more	73 (36%)	28 (39%)	21 (30%)	24 (38%)	

^a^Registered nurses, registered practical nurses, or personal support workers. ^b^BSO leads are mostly front-line nursing staff, but can occasionally be allied health clinicians. ^c^ Row excluded from chi-sqaured analysis due to small cells

### Barriers and facilitators regarding isolation and quarantine

LTCH staff identified that residents’ cognitive impairment (94%, 95% CI 90-96%) and language/communication barriers (67%, 95% CI 60-73%) were the most common challenges faced when trying to isolate them. Almost all respondents observed that residents who were meant to be isolated entered common areas (90%, 95% CI 85-93%) or other residents’ rooms (85%, 95% CI 79-89%), and residents did not follow hand hygiene/masking guidelines (81%, 95% CI 75-86%). Staff found it challenging to mitigate the mental health impacts of isolation, primarily resident loneliness (90%, 95% CI 86-94%) and boredom (88%, 95% CI 83-92%). The vast majority of respondents observed a decline in physical and/or emotional well-being of residents (84%, 95% CI 78-88%) attributed to isolation. Two-thirds identified issues with resident safety while alone in their rooms (68%, 95% CI 61-74%), and a similar proportion reported verbal aggression (78%, 95% CI 72-83%) or physical aggression (61%, 95% CI 54-67%) towards staff by residents responding to the direction to stay in their rooms. Staff also observed resident anxiety or fear (66%, 95% CI 59-72%) and at times the development of paranoia (46%, 95% CI 39-52%) in response to the isolation.

**Fig. 1:**
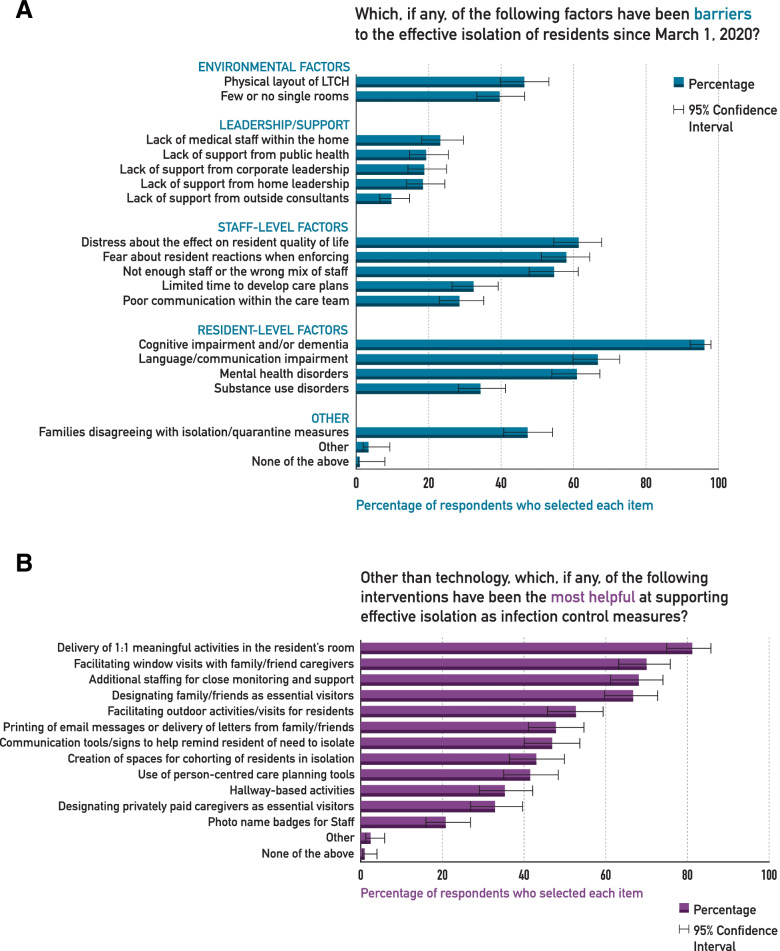
Barriers (**A**) and Facilitators (**B**) to the isolation or quarantine of long-term care residents

Figure 1 A illustrates the barriers to effective isolation of residents as reported by LTCH staff. Respondents placed an emphasis on resident-related factors such as cognitive impairment and mental health disorders, and to a lesser extent on the leadership and supports received during the pandemic. Almost two-thirds (61%, 95% CI 55-68%) identified that staff distress about the effects of the ICP measures on residents’ quality of life was a barrier to their implementation, and 59% (51-65) were fearful of residents’ reactions when trying to enforce the measures. Respondents rated a number of different strategies as valuable in supporting effective isolation (Fig. 1B). The most effective strategies identified were engagement of the residents in meaningful activities, providing close monitoring, and facilitating different kinds of visits from families/friends.

**Fig. 2:**
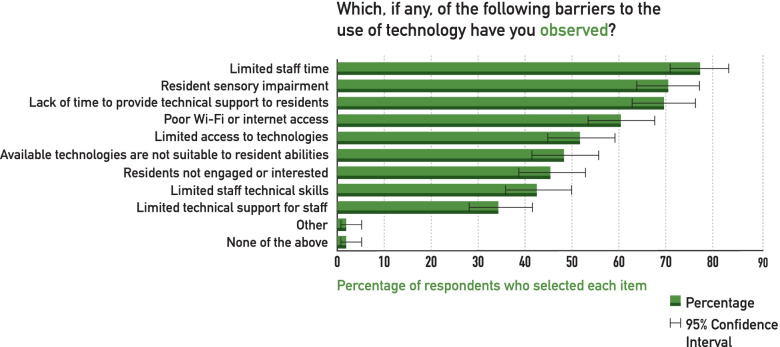
Barriers to the use of technology to support long-term care home residents during isolation/quarantine

Technologies were separately rated in how helpful they were in mitigating the impact of isolation. Video calling of family and friends was found to be the most helpful, with 89% (95% CI 83-92%) reporting it as moderately or more helpful. Door and bed alarms were rated moderately or more helpful by 51% (95% CI 44-58%), followed by video calling with healthcare providers (49%, 95% CI 42-56%), video volunteer “friendly visitors” (34%, 95% CI 27-40%), and individualized virtual activities (34%, 95% CI 27-40%). Few respondents found technology was helpful to monitor residents in their rooms, such as with video monitors (19%, 95% CI 14-25%) or location tracking technologies (12%, 95% CI 8-18%). The most common barriers to the use of technology in supporting residents during isolation are illustrated in Fig. 2, with lack of time (77%, 95% CI 71-83%), sensory impairment (71%, 95% CI 64-76%), and poor Internet connectivity among the most important barriers (60%, 95% CI 54-67%).

### Dementia Isolation Toolkit

**Fig. 3:**
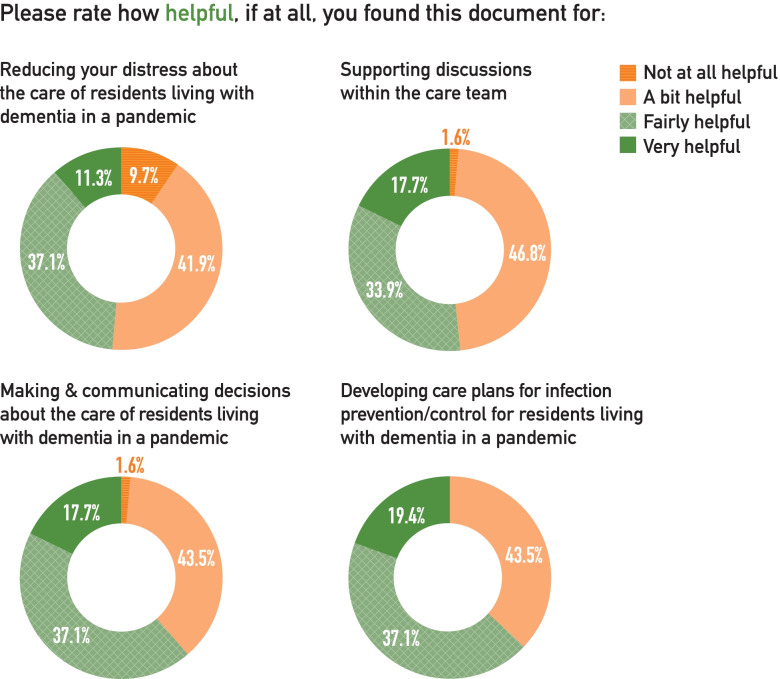
Ratings of the helpfulness of the DIT by those respondents who had used it

135 (65%) respondents identified themselves as at least “a bit familiar” with the DIT, with 62 (30%) saying they were fairly or very familiar with the toolkit. Within the group of those who were familiar with the toolkit, 65 (48%) used this resource to guide decision-making about care. Of those who used the toolkit, 40 (62%) found it fairly or very helpful. Around two-thirds of respondents who used the DIT found it fairly or very helpful for developing isolation care plans (63%) or making and communicating decisions about care (61%; Fig. 3). About half (48%) reported that the DIT was fairly or very helpful at reducing their distress in providing care during the pandemic. The elements of the DIT toolkit rated as most helpful were the worksheet for person-centred isolation care planning (74%), followed by information about the risks and benefits of different approaches to isolation (71%) and how to apply ethical principles in decision-making (60%), and the decision-making worksheet (50%).

Between the three groups of respondents (Table 1), the most notable difference was that BSO leads were much more likely to have used the DIT (Pearson’s chi2= 31.5; RRR 9.1 (95% CI 2.8-29.7); p < 0.001). Men were less likely to be familiar with the DIT (Pearson’s chi2= 6.8; RRR 0.1 (95% CI 0.01-0.8); p = 0.034) and increasing years of LTC experience increased the odds of being familiar with the DIT (Pearson’s chi2= 18.5; OR 2.0 (95% CI 1.1-3.7); p = 0.022). There were no differences between groups in LTCH characteristics.

Moral distress was common across the survey respondents with one-third (33%) reporting large to extreme amounts of moral distress, 84% reporting an increase in moral distress since the start of the pandemic, and 40% noted a large impact of moral distress on their job satisfaction (Table 2). Overall, there were no differences in moral distress between those who were familiar with or used the DIT compared to those who were unfamiliar with it (Table 2). However, after controlling for differences between groups in role, gender and years of experience, those who used the DIT reported less impact of moral distress on job satisfaction (Table 3; OR 0.41 (95% CI 0.19-0.87); p = 0.019).

**Table 2:** Moral distress reported by respondents by group

	Total respondents (N = 207)	Not familiar with DIT (n = 72)	Familiar with the DIT (n = 70)	Used the DIT (n = 65)	Pearsons chi2 (p-value)
**Amount of Moral distress**					2.3, *df*=8 (0.971)
None at all	16 (8%)	4 (6%)	6 (9%)	6 (9%)	
A small amount	51 (25%)	15 (21%)	20 (29%)	16 (25%)	
A moderate amount	70 (34%)	25 (36%)	23 (33%)	22 (34%)	
A large amount	47 (23%)	19 (27%)	14 (20%)	14 (22%)	
An extremely large amount	20 (10%)	7 (10%)	7 (10%)	6 (9%)	
**Change in Moral distress**					9.4, *df*=8 (0.311)
Significantly decreased	2 (1%)	1 (2%)	0	1 (2%)	
Somewhat decreased	6 (3%)	1 (2%)	3 (5%)	2 (3%)	
About the same	21 (11%)	11 (17%)	4 (6%)	6 (10%)	
Somewhat increased	92 (49%)	27 (42%)	31 (48%)	34 (59%)	
Significantly increased	66 (35%)	25 (38%)	26 (41%)	15 (26%)	
**Moral distress impacting job satisfaction**					12.0, *df*=8 (0.149)
Not at all	19 (9%)	9 (14%)	7 (11%)	3 (5%)	
A small amount	42 (20%)	10 (15%)	18 (26%)	14 (22%)	
A moderate amount	45 (22%)	15 (23%)	12 (17%)	18 (28%)	
A large amount	49 (24%)	15 (23%)	18 (26%)	16 (25%)	
An extremely large amount	32 (15%)	17 (26%)	9 (13%)	6 (9%)	

**Table 3:** Relationship between components of moral distress and familiarity with or use of the DIT

Components of moral distress	DIT exposure	Unadjusted OR (95% CI)	Adjusted for role OR (95% CI)	Adjusted for role, gender, years of experience OR (95% CI)
Amount of moral distress	Not familiar	1.0	1.0	1.0
	Familiar	0.70 (0.39-1.3)	0.70 (0.38-1.3)	0.67 (0.35-1.3)
	Used	0.74 (0.40-1.4)	0.74 (0.38-1.4)	0.72 (0.36-1.4)
Change in moral distress	Not familiar	1.0	1.0	1.0
	Familiar	1.3 (0.67-2.5)	1.3 (0.65-2.6)	1.3 (0.63- 2.7)
	Used	0.75 (0.38-1.5)	0.60 (0.28-1.3)	0.62 (0.29-1.4)
Moral distress impact on reduced job satisfaction	Not familiar	1.0	1.0	1.0
	Familiar	0.67 (0.36-1.2)	0.54 (0.28-1.0)	0.48 (0.24-0.98)*
	Used	0.73 (0.39-1.4)	**0.46 (0.23-0.92)†**	0.41 (0.19-0.87)**

**†**p = 0.028; *p = 0.045; **p = 0.019

### Free-text responses

 A subset of respondents (n = 116) provided free text responses (total units=239) about which resources and supports were helpful or would be helpful to support isolation of residents. Their responses were grouped into three overarching categories: Resources (n = 92); Teamwork (n = 63); and Activation (n = 84). Under resources, by far the most frequent theme was the need for sufficient staff to support and monitor the residents (n = 42):

*“In short, the more PEOPLE, the better. In the absence of most family members, caregivers, volunteers and large group gatherings/events, the presence of additional staff to visit one-to-one, do small group activities, help make phone calls and host skype chats - this has made the biggest difference”.*

Under ‘Resources’, technology was the next most frequent theme (n = 26), with respondents describing the benefits of technology to their residents, while highlighting cost and lack of WiFi as important barriers. Financial constraints were raised (n = 8), particularly the need *“to have a budget to be able to buy things for residents.*” Also mentioned were funds to buy personal televisions or tablets, to hook up cable TV in residents’ rooms, or buy “*practical individualized activity kits.*”

Under ‘Teamwork’, the importance of various internal (n = 9) and external (n = 25) supports to the LTCH were emphasized, as was the importance of having *“fully engaged staff from all departments with the resident as their primary focus on your team*.” They also highlighted the effect of disruptions to the team, including the use of temporary or agency staff (e.g., “*dumping in more bodies that are not yet part of the institution’s ‘team’ does not always work out well*”) or attitudes (e.g., “*staff not willing to trial creative interventions*”). Respondents wrote about the need for good communication (n = 11), e.g.,*“Communication, communication, communication, to your staff, your families and your residents keeps everyone up to date and comfortable with the decisions that the home is making”.* Finally, the importance of family as part of the team (n = 8) was emphasized (e.g., *“Keeping loved ones connected has proven to be largest benefit”).*

Regarding ‘Activation’, a large number of the comments addressed the importance of activity and recreation for supporting isolated residents. Activities mentioned often included outdoor walks, music, virtual church services, as well as the need to adapt activities to the pandemic. For example: *“Recreation department has come up with some innovative ways to engage residents. hallway bingo, dart league, craft carts, magazine carts, garden cart. Items were disinfected afterwards”.* An important theme was the need for more specialized recreation staff (n = 15). For example:*“There is a shortage when it comes to nursing but … an increase in activity staff is extremely beneficial. They approach the resident’s isolation from a different point of view”.*

Two further themes within this category of Activation were the need for 1:1 engagement with isolated residents (n = 28), and the need for the activity to be personally meaningful for the resident (n = 12), e.g. *“Knowing your residents, what makes them happy and providing that, to the best of your ability, even while having to keep them within the confines of their room is key”.*

## Discussion

LTCH staff find it difficult to implement isolation as an ICP practice given the nature of the residents, the environment, and the resources available in long-term care. While this difficulty has been identified in the context of other respiratory viral outbreaks and multi-drug resistant bacteria in LTCH [25–28], it has been brought to the fore by the COVID-19 pandemic. The need for isolation for infection control, and the difficulties in doing so effectively and compassionately, creates a number of ethical dilemmas for staff in long-term care. As the COVID-19 pandemic evolved, it became clear that navigating these ethical dilemmas required some degree of knowledge or understanding about public health ethics, particularly where they differ from the usual medical ethics that guide clinical care [29]. Most LTC staff had not received such training, and this was the impetus for the development of the DIT.

In line with other surveys of LTCH during the pandemic, we found that people working in LTCH felt ill-prepared and under-resourced, particularly where staffing was concerned [30–32]. Across front-line, administrative, and medical staff in our survey, there was consensus that there were not enough staff to support residents to remain safely in their rooms. Even outside of the pandemic, lack of resources is a common source of ethical challenge in LTCH [33]. LTCH residents have increasingly complex needs [34], including high rates of dementia and mental health disorders, and the failure of the aging LTCH infrastructure to address these needs is particularly apparent during outbreaks [35]. Going beyond the resource and infrastructure barriers, respondents also strongly endorsed barriers related to their own emotional experience of isolating residents. For example, respondents reported that their own distress about needing to isolate residents, and their anxiety about how the residents would react if they tried to enforce these rules, had an important impact on the effectiveness of isolation. This is in keeping with previous research demonstrating that staff are acutely aware of the conflict between the infection control protocols in LTCH and resident quality of life goals [36–39]. Despite this, no tools or strategies have previously been developed to help staff manage this conflict and thus effectively and compassionately apply infection control precautions.

It was clear from the survey that much effort went into finding creative ways to support resident engagement during the pandemic. Programming shifted from group activities to 1:1, and the use of video-calling and consultation expanded enormously [40]. While technology has been found to be a promising tool, staff also enumerated some significant barriers to its use, in particular the costs of equipment suitable for the resident population and Internet access. There is a sizable group of residents of LTCH who are indigent and thus do not have access to funds for basic items that would improve their quality of life, such as televisions or tablets. The digital disenfranchisement of marginalized groups is an important issue emerging from this pandemic [41]. In the context of LTCH, there is a need to urgently accelerate the provision of adequate Internet access to support resident social connections and engagement, particularly during outbreak periods [42].

As our sample was not random, we are unable to speak to the overall degree of uptake of the DIT in LTCH in Ontario. However, we did find that the DIT was largely disseminated and implemented using the structure of BSO [43]. BSO is a provincial agency that works with LTCH to provide enhanced health care services for older adults with responsive behaviours. The existing structure of BSO includes practice leads embedded within LTC with specialized skill and knowledge in working with people living with dementia, protected time for training, and access to a well-established provincial knowledge dissemination network. This proved to be a model for fostering dialogue about how to address the challenges being faced and for sharing best practices. Overall, this speaks to the value of having dedicated and skilled resources embedded within LTCH and the value of just-in-time learning models during periods of rapid practice change [44].

While moral distress of staff is known to be endemic in the under-resourced LTCH sector [45, 46], our results provide some initial evidence for dramatic increases in moral distress in LTCH staff over the pandemic period. To address moral distress, the best available evidence points to interventions that are experiential and incorporate ethics education, structured ethical reflection, and opportunities to discuss conflicts within the care team [47–50]. While our survey was not designed to specifically evaluate the effectiveness of the DIT, we found some preliminary evidence that, after controlling for difference between groups, those who used the DIT reported less impact of moral distress on their job satisfaction. About half of those individuals who used the DIT also reported that it was helpful for reducing distress. While a more comprehensive evaluation of the impact of implementing the DIT on moral distress is currently underway,[51] previous research in LTCH has found some important facilitators for engaging LTC staff in practice change involving ethics work. These include support from leadership and embedding opportunities for ethical reflection and discussion in daily work [18, 49, 52, 53]. At present, while ethics committees or reflection groups are becoming more common in LTCH settings, they are rarely used for everyday ethical decision-making, and tend to focus on end-of-life decision-making [49, 54]. The pandemic, while catastrophic for LTCH, may have also created the conditions for greater uptake and engagement in ethics as part of the daily work of LTCH staff, which hopefully can be carried forward beyond the pandemic.

A strength of this study was the diverse sample of LTCH staff from across the province of Ontario, including rural and urban, large and small LTCH, and for profit and not-for-profit. An important limitation is that the sample was not random and was likely enriched by those who were motivated to comment on this issue, and by those with some familiarity with DIT. This makes it difficult to generalize our findings to the wider population of people working in LTCH. There is no information available to us about the characteristics of this large workforce to allow us to establish whether our sample is broadly representative. While it was possible to complete the survey multiple times, the lack of a direct incentive and the duration of the survey (average of 23 min) makes this risk low. To maintain the anonymity of the survey, we did not collect the names of LTCH, thus we cannot establish if there was any clustering by facility, although we believe this is also low risk. There are over 600 LTCH in Ontario, and the link was distributed through province-wide networks, not through individual LTCH. There was a broad representation of facility characteristics, and none were associated with DIT familiarity.

## Conclusions

 There are many barriers to the safe and effective implementation of isolation as an ICP measure in the LTCH environment. LTCH staff identify that these measures can be harmful to residents and cause moral distress. There is a role for dementia-specific ethical guidance, such as the DIT, to support best ICP practices that address the unique needs of LTCH residents.

## Supplementary Information

**Additional file 1.**

**Additional file 2.**

## Data Availability

The survey data used in this study is provided in an aggregate form as supplementary material. The raw survey datasets generated and/or analysed during the current study are not publicly available as they contain information which could be used to re-identify participants, but the data are maintained in an institutional data repository and are available from the corresponding author on reasonable request.

## Abbreviations

LTCH: Long Term Care Home
ICP: Infection Control and Prevention
DIT: Dementia Isolation Toolkit
BSO: Behavioural Supports Ontario
