# Supplementary material for: Barriers and facilitators to person-centred infection prevention and control: results of a survey about the Dementia Isolation Toolkit
Source: BMC Geriatr. 2022 Jan 25;22:74. doi: 10.1186/s12877-022-02759-4 (PMC8786624; doi:10.1186/s12877-022-02759-4)
Supplement: Supplementary file 1 — Additional file 1. [file 12877_2022_2759_MOESM1_ESM.pdf]

Appendix A: Survey Tool

Dementia Isolation Toolkit Survey

Consent:

You are being asked to participate in this survey given your role as a staff member and/or administrator of a long-term care home (LTCH) in Ontario during the COVID-19 pandemic. Depending upon the depth of your responses, the survey takes an average of 20 minutes to complete.

The study is being conducted by researchers who are part of the Dementia Isolation Toolkit Working Group, based at KITE-Toronto Rehabilitation Institute, University Health Network, and led by Dr. Andrea Iaboni. This study is funded by the Ontario COVID-19 Rapid Research Fund. The aims of this survey are to understand the impact of infection control measures on the care of people with dementia living in LTCH and on the healthcare professionals in LTCH, and to evaluate and improve the Dementia Isolation Toolkit. We will be asking questions about your role in LTCH, your experiences working in LTCH through the COVID-19 pandemic, your use of the Dementia Isolation Toolkit if applicable, and some personal demographic information. The results of the completed surveys will be used to inform the ongoing development of the Dementia Isolation Toolkit used to support LTCH and people living with dementia in LTCH through the COVID-19 Pandemic and future pandemics or outbreaks.

Your participation in this study is completely voluntary. There is a risk that you may feel uncomfortable answering some of these questions. However, you may skip any questions you do not wish to answer, and you can withdraw from the survey at any point without any consequences. Please know that once you submit the survey, it will not be possible to withdraw your responses as the survey is anonymous. If you decide to participate, please complete the questions to the best of your ability. It is very important for us to learn about your experiences. You will not be compensated for participating in this survey, but you can choose to be included in a draw for one of five

$100 gift cards. Your odds of winning a prize is based on the number of individuals who participate in the study. Information collected to draw for the prizes will not be linked to the study data in any way, and this identifying information will be stored separately, then destroyed after the prizes have been provided.

The online survey data is being collected by the Survey Research Centre (SRC) at the University of Waterloo on behalf of the researchers. The survey data collected will be summarized and anonymized so that no individual can be identified from these summarized results. We may use quotes from your open-ended survey responses in summary reports, however, these quotes will be anonymous. The survey responses will be stored on a restricted access, secure server at the University of Waterloo and transferred by the SRC to a secure server at the University Health Network and electronically archived for at least seven years. When information is transmitted over the internet, privacy cannot be guaranteed. There is always a risk your responses may be intercepted by a third party (e.g., government agencies, hackers). The SRC temporarily collects your computer IP address to avoid duplicate responses in the dataset.

If you have any questions about the research study, please contact the study research coordinator, Hannah Quirt at: Hannah.Quirt@uhnresearch.ca

This study has been reviewed and received ethics clearance by the University Health Network Research Ethics Board (REB#20-5866) and a University of Waterloo Research Ethics Committee (ORE#42803). If you have any questions about your rights as a research participant or have concerns about this study, you may call the University of Waterloo Office of Research Ethics at 1-519-888-4567 ext. 36005 or email:ore-ceo@uwaterloo.ca.

Please note that communication via email is not absolutely secure. Thus, please do not communicate personal sensitive information via email.

I consent to participate in this survey. By providing your consent, you are not waiving your legal rights or releasing the investigator(s) or involved institution(s) from their legal and professional responsibilities.

Yes **GO TO NEXT SECTION**
No **GO TO END OF SURVEY**

DEMENTIA ISOLATION TOOLKIT WEB SURVEY

We would like to first ask you a few questions about <u>your role at the long-term care home (LTCH) you work in.</u> If you work in more than one LTCH, think about one in which you work **most often.**

A1 What is your role in your LTCH?: (select one)

01Administrator
02 Director of Care/Director Residential Care
03 Assistant Director of Care
04 Nurse Manager
05 Registered Nurse/Registered Practical Nurse/Licensed Practical Nurse
06 Behavioural Supports Ontario (BSO) Team Member
07 Recreation/Life Enrichment
08 Social Worker/Social Service Worker
09 Occupational Therapist (OT)
10 Physical Therapist (PT)
11 Behaviour Therapist
12 Dietary Staff
13 Environmental Services Staff
14 Other, please specify: ______________________

A2 How many LTCH spaces/beds does this LTCH currently have?

01 <50 beds
02 50-99 beds
03 100-149 beds
04 150-199 beds
05 200-249 beds
06 >250 beds

A3 What is the population of the city/town where this LTCH is located?

01 Rural to small (less than 30,000 population)
02 Medium (30,000 to less than 100,000 population)
03 Large urban (more than 100,000 population)

A4 What is the ownership status of this LTCH?

01 Government-owned (municipal)
02 Not-for-profit
03 For-profit
04 I do not know

A5 Since March 1, 2020 (start of COVID-19 pandemic and restrictions), has it been more or less challenging to ensure adequate staffing levels in this LTCH?

01 Significantly more challenging
02 Somewhat more challenging
03 About the same
04 Somewhat less challenging
05 Significantly less challenging

A6a How many outbreaks, if any, has this LTCH experienced?

01 None (skip to A7)
02 1
03 2
04 3
05 4 or more

A6b What is the current status of the most recent outbreak?

01 Currently resolved
02 Currently active

How large was/is the largest outbreak in this home?

A6c Among residents:	A6d Among staff
01 No residents	01 No staff
02 Less than 5 residents	02 Less than 5 staff
03 5-9 residents	03 5-9 staff
04 10-20 residents	04 10-20 staff
05 More than 20 residents	05 More than 20 staff
06 I don’t know	06 I don’t know

A7 In your opinion, thinking back to just prior to March 1, 2020, how prepared do you think your LTCH was for a COVID-19 outbreak?

01 Not at all prepared
02 Poorly prepared

03 Somewhat prepared
04 Well prepared
05 Very well prepared

……….

In this section, we are interested in your perspectives and experiences on barriers and facilitators regarding the implementation of infection control measures for residents with dementia in LTCH. We are interested in any measure that involves asking a resident to remain in their room to prevent the spread of infection. The word **isolation** is used to refer to both isolation and quarantine.

B1 Have you participated in any way (directly or indirectly) in the isolation or quarantine of residents since March 1, 2020?

01 No
02 Yes

If no, skip to section C

B2 Which, if any, of the following factors have been barriers to the effective isolation of residents since March 1, 2020? (select all that apply)

01 Few or no single rooms
02 Physical layout of LTCH impacting ability to cohort residents
03 Lack of guidance and support from LTCH leadership in the home
04 Lack of guidance and support from LTCH leadership at the corporate level
05 Lack of guidance and support from public health
06 Lack of guidance and support from outside consultants or specialists
07 Lack of medical staff within the home
08 Staff distress about the effect of measures on resident quality of life
09 Staff fear about resident reactions when enforcing the need for them to remain in their room
10 Families expressing disagreement or concern with isolation or quarantine measures
11 Poor communication within the care team
12 Limited time to develop care plans
13 Not enough staff or the wrong mix of staff (for example, no senior staff or staff familiar with unit present)
14 Delays in putting infection control measures in place (i.e. lack of timely action by staff)
15 Resident cognitive impairment and/or dementia (e.g. unable to remember need to stay in room)
16 Resident impairments in language and/or communication

17 Residents with mental health disorders (e.g. schizophrenia or bipolar disorder)
18 Resident substance use disorder (e.g. smoking, alcohol)
19 Other, please specify:
20 None of the above

B3 Which, if any, of the following situations or challenges related to isolation have you encountered? (select all that apply)

01 Residents unable to understand or appreciate the need for isolation or quarantine
02 Residents who understand the need for the infection control measures, but who refuse to comply
03 Residents leaving their room to go into common areas
04 Residents leaving their room and entering other residents' rooms
05 Residents trying to leave their unit or building
06 Residents not wearing masks or washing hands
07 Residents engaging in unhygienic behaviours outside of their rooms (e.g. spitting, touching many surfaces in the LTCH)
08 Residents exhibiting anxiety, fear or paranoia about the request to stay in their room
09 Residents responding verbally in an aggressive way towards staff trying to direct them back to their rooms
10 Residents responding physically in an aggressive way towards staff trying to direct them back to their rooms
11 Residents potentially being unsafe when alone in their room (e.g. trying to get up from bed without assistance, smoking)
12 Residents exhibiting a decline in physical and/or emotional well-being with isolation or quarantine
13 Residents developing delusional beliefs related to isolation (e.g. that their family members have died or moved away).
14 An increase in conflict between residents in shared rooms
15 Residents experiencing loneliness and/or other effects of separation from families/other residents
16 Residents experiencing boredom or a lack of stimulation
17 Other, please specify:_________
18 None of the above

B4 How much, if at all, have technology-based approaches or strategies helped mitigate the impact of COVID-19 isolation on residents in your LTCH?

	Not at all	A small amount	A moderate amount	A large amount	An extremely large amount	Not Applicable
01 Use of technology to monitor resident in room (e.g. baby monitors, two-way video monitors)						
02 Alarms (bed or door)						
03 Real-time location tracking systems						
04 Video calling with resident's family or friend caregivers						
05 Video calling with resident's physician(s) or other healthcare providers						
06 Video calling with volunteer friendly visitor programs and/or spiritual/religious leaders						
07 Individualized Virtual Activities (e.g. exercise programs, use of Memory Care Tablet Apps)						
08 Other, please specify:						

B5 Which, if any, of the following barriers to the use of technology have you observed? (select all that apply)

01 Poor Wi-Fi or internet access
02 Limited access to technologies such as tablets for staff
03 Limited time for staff to make use of available technologies
04 Limited staff technical or computer skills
05 Limited technical support for staff
06 Lack of staff time to provide technical support to residents
07 Residents do not seem engaged or interested in the available technologies
08 The available technologies are not suitable based on the cognitive abilities of the residents
09 Resident sensory impairment (e.g. vision, hearing)
10 Other, please specify:
None of the above

B6 Other than technology, which, if any, of the following interventions have been the most helpful at supporting effective isolation as infection control measures (select all that apply)

01 Additional staffing for close monitoring and support

02 Creation of spaces for cohorting of residents in isolation/quarantine
03 Communication tools/signs to help remind resident of need to isolate
04 Use of person-centred care planning tools (e.g. P.I.E.C.E.S.™ Framework or Dementia Isolation Toolkit)
05 Designating family/friend as essential visitors to support isolation in person
06 Designating privately paid caregivers as essential visitors to support isolation in person
07 Facilitating window visits with family/friend caregivers
08 Facilitating outdoor activities / visits for residents (including with family/friend caregivers)
09 Delivery of 1:1 meaningful activities in the resident's room
10 Hallway-based activities (whereby residents participate from their doorways)
11 Printing of email messages or delivery of letters from family/friends
12 Photo name badges for Staff
13 Other, please specify: __________
14 None of the above

B7 What is your level of familiarity with the Dementia Isolation Toolkit? [http://dementiaisolationtoolkit.com] (see picture)

01 Not at all familiar (skip to B10)
02 A bit familiar
03 Fairly familiar
04 Very familiar

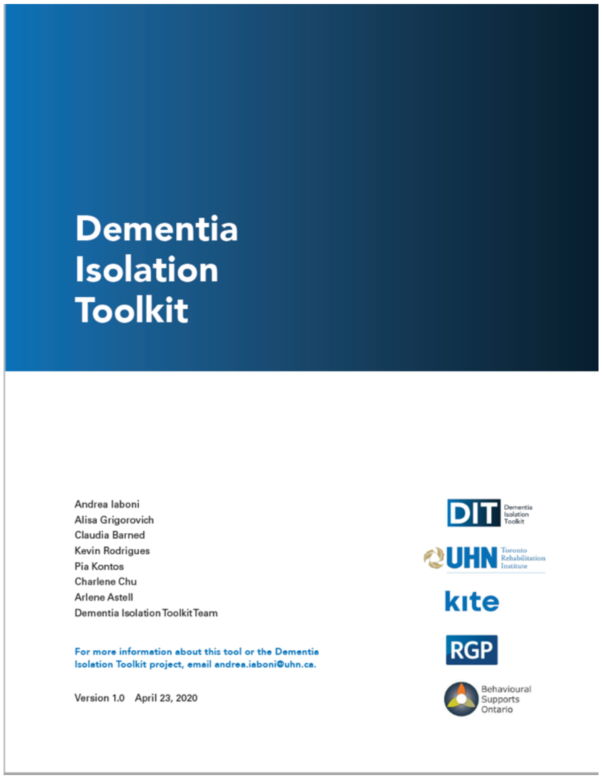

B8 Have you seen the document about ethics and person-centred isolation care planning as part of the Dementia Isolation Toolkit?

01 No (skip to B9)
02 Yes

B8a Did you see the Dementia Isolation Toolkit document about ethics and person-centred isolation care planning before or after a COVID-19 outbreak at your LTCH?

01 Before
02 During or After
03 N/A—there has not been an outbreak at my LTCH
04 I don't know

B8b. Have you used information from the Dementia Isolation Toolkit document to help guide decision-making about the care of residents during the COVID-19 pandemic?

01 No (skip to B9)
02 Yes

B8c. Please rate how helpful, if at all, you found this document for:

	Not at all helpful	A bit helpful	Fairly helpful	Very helpful
01 Developing care plans for infection prevention/control for residents living with dementia in a pandemic				
02 Making & communicating decisions about the care of residents living with dementia in a pandemic				
03 Reducing your distress about the care of residents living with dementia in a pandemic				
04 Supporting discussions within the care team				

B8d. What elements, if any, of the Dementia Isolation Toolkit were the most helpful? (Select all that apply)

01 Information about the ethical principles that are important in a pandemic
02 Information about how to apply ethical principles when making decisions about isolation care in a pandemic
03 Information about the risks and benefits of different approaches to isolation care in a pandemic
04 Information about how to make ethical decisions in a pandemic
05 Worksheet for person-centred isolation care planning
06 Decision-making worksheet
07 None of the above

B9 What information, if any, do you think would be the most helpful to add to the Dementia Isolation Toolkit? (Select all that apply)

01 Practical information about how to deal with ethical dilemmas you may face in your work
02 Case studies that show how the worksheets can be used for isolation care planning and decision-making

03 A legal framework that explains the relationship between different laws and regulations and how they govern the use of isolation and quarantine as infection control measures
04 Tools focused on educating and supporting families
05 Information on how technology can be used to support residents in isolation
06 Practical tips and tricks in supporting residents in isolation
07 Other, please specify:
08 None of the above

B10 What other resources or supports have you found helpful to support the isolation or quarantine of residents living with dementia?

[open-ended]

B11 What additional resources or supports would be helpful to support the isolation or quarantine of residents living with dementia?

[open-ended]

……………………..
In this section, we are interested in your perspectives and experiences regarding the impact of COVID-19 (and related restrictions) on the care of residents with dementia:

C1 In your opinion, since March 1, 2020, how has COVID-19 and related infection control measures affected the quality of care for residents living with dementia in your LTCH? The quality of care is:
01 Significantly worse
02 Somewhat worse
03 About the same
04 Somewhat improved
05 Significantly improved

C2 Since March 1, 2020, how have infection control measures affected the frequency and severity of responsive behaviours among residents living with dementia in your LTCH? These behaviours are:
01 Significantly worse
02 Somewhat worse
03 About the same
04 Somewhat improved
05 Significantly improved

C3 Based on your observations, how has the health and well-being of residents with dementia been affected by the COVID-19 pandemic and infection control measures?

	**Significantly worse**	**Somewhat worse**	**About the same**	**Somewhat Improved**	**Significantly Improved**	**I do not know**
Cognitive function						
Daily functions (e.g. ability to eat, dress, bathe, transfer, toilet, hygiene care)						
Mobility						
Food and fluid intake						
	**Significantly increased**	**Somewhat increased**	**About the same**	**Somewhat decreased**	**Significantly decreased**	**I do not know**
The presence of pressure ulcers						
The frequency of falls						
The use of medications for responsive behaviours						
The use of physical restraints						

C4 Please describe any other important ways the COVID-19 pandemic and related infection control measures have impacted the well-being of residents living with dementia and their care.

[open-ended]

In this section, we would like to ask you a few questions about yourself and the impact of COVID-19 on your working life and well-being:

D1 Which age group do you belong to?

01 Under 18
02 18-34
03 35-44
04 45-54
05 55 or older

D2 What best describes your gender?

01 Female
02 Male
03 Non binary/gender diverse
04 Prefer to self-describe: __________
05 Prefer not to answer

D3 What is your ethnic or cultural identity? (select all that apply)

01 White
02 South Asian (e.g., East Indian, Pakistani, Sri Lankan, etc.)
03 Chinese
04 Black
05 Filipino
06 Latin American
07 Arab
08 Southeast Asian (e.g., Vietnamese, Cambodian, Laotian, Thai, etc.)
09 West Asian (e.g., Iranian, Afghan, etc.)
10 Korean
11 Japanese
12 First Nations, Inuit or Métis
13 Other, please specify: __________
14 Prefer not to answer

D4 Which of the following professional qualifications do you have? (select all that apply)

01 PSW / HCAs (Personal Support Worker / Health Care Aides)
02 RPN (Registered Practical Nurse)
03 RN / BScN / BN (Registered Nurse/Bachelor of Science in Nursing/Bachelor of Nursing)
04 Nurse Practitioner
05 MN (Masters of Nursing)
06 MSW (Masters of Social Work)
07 MPH/MSPH (Masters in Public Health; Health Administration)
08 Other, please specify: __________
09 Prefer not to answer

D5 How many years of experience do you have working in any LTCH?

01 Less than 5 years
02 6-10 years
03 11-15 years
04 16-20 years
05 More than 20 years

D6 Since March 1, 2020, on average, how many days per week have you spent providing or supporting care in person at a LTCH?

01 Less than 2 days per week
02 2-4 days per week
03 More than 4 days per week

D7 The following questions are about "moral distress". Moral distress is the bad feelings a healthcare professional gets when they know the right thing to do for a resident, but for some reason, is unable to do it.

In these questions you will be asked about your level of distress, if any, resulting from different situations related to the COVID-19 pandemic. First, consider whether you have found yourself in this situation since March 1, 2020. If not, check the "Not applicable" box and move to the next item. If you have found yourself in this situation, indicate how much that situation bothered you or distressed you.

	Since March 1, 2020, how much distress, if any, have the following situations caused for you?					
	N/A Has not happened to me since March 1, 2020	None at all	A small amount	A moderate amount	A large amount	An extremely large amount
1. Seeing the care suffer for residents with dementia because there are not enough staff to do the work.						
2. Seeing a low quality of life for residents with dementia because there are not enough activities.						
3. Seeing the care suffer for residents with dementia because of high staff turnover or new staff without the training to provide dementia care.						
4. Having to provide care to aggressive residents with dementia without the supports I need to feel safe.						
5. Seeing the care suffer for residents with dementia because physicians do not visit often enough						
6. Seeing the care suffer for residents with dementia because of the effects of restrictions on family visits						
7. Having to follow COVID-19 related policies or procedures even when they don't seem best for the residents						
8. Seeing residents with dementia suffering from pain or other symptoms because they are not treated appropriately.						
9. Not reporting what I believe is neglect or abuse of a resident						

with dementia because I feel no one listens or I'm afraid of causing trouble.						
10. Seeing poor care for a resident with dementia because of poor communication between staff members.						

D8 Overall, how much, if any, moral distress do you currently feel in your job?

01 None at all (skip to D12)
02 A small amount
03 A moderate amount
04 A large amount
05 An extremely large amount

D9 Overall, how has the amount of moral distress you experience in your work changed since the start of the pandemic?

01 Significantly decreased
02 Somewhat decreased
03 About the same
04 Somewhat increased
05 Significantly increased

D10 How often, if ever, have you felt the following as a result of the moral distress of caring for residents with dementia in LTCH since March 1, 2020?

	Never	At least once	Once a month or more	Once a week	More than once a week
Feel frustrated or angry					
Feel guilty or like a failure					
Feel powerless					
Feel sad or anxious					
Not want to go to work					
Lose sleep					
Feel physically exhausted					
Feel sick or in physical pain					
Do things I know are not healthy to cope (e.g. overeating, drinking, smoking, etc.)					

D11 As you answer the two questions below, please think about how moral distress affects how you feel about your job.

	Not at all	A small amount	A moderate amount	A large amount	An extremely large amount
1. How much does moral distress reduce my job satisfaction?					
2. How much does moral distress make me want to quit my job?					

D12 Reflecting on the overall impact of COVID-19 and various restrictions in the LTCH where you work, is there anything else you would like us to know about your experience of caring for with residents living with dementia?

01 No
02 Yes, please specify: ___________

E1 We may wish to contact you within the next three months to take part in a follow-up telephone interview. This interview would take about 45 minutes and we will provide a gift card of $30.00 to thank you for your time. Would you be willing to let us contact you again about a follow-up interview? This information will be stored separately from your survey responses to maintain the anonymity of the survey.

01 No (skip to E2)
02 Yes (direct to separate web page)

Future research page

Please provide your contact information below. This information will be stored separately from your survey responses to maintain the anonymity of the survey.

First Name:
Last Name:
Phone Number (with area code):

E-mail address:
E-mail address (confirmation):

E2 For your participation, would you like to be entered into a draw for one of five $100 gift cards? This information will be stored separately from your responses to maintain the anonymity of the survey and will only be used to contact you to distribute the gift card.

01 No -- **GO TO THANK YOU PAGE**
02 Yes -- **GO TO DRAW ENTRY PAGE**

Draw Entry Page

The draw winners will be selected and notified once the survey closes. The amount received is taxable. It is your responsibility to report this amount for income tax purposes.

First Name:
Last Name:
Phone Number (with area code):
E-mail address:
E-mail address (confirmation):

Thank You Page

That is the end of the survey. On behalf of the researchers, we would like to thank you very much for your time. Once the study is complete, general anonymized findings will be combined and reported in summary research reports and publications. You may contact the principal investigator [Dr. Andrea Iaboni at Andrea.iaboni@uhn.ca] or study coordinator [Hannah Quirt at: Hannah.Quirt@uhnresearch.ca] if you wish to obtain a summary report of the study.

Please note if you have any concerns about the care provided to a resident in a LTC home, please inform the Director of Care and/or call the Ontario Long-Term Care Homes ACTION line: toll-free 1-866-434-0144.
